# SiFBA5, a cold-responsive factor from *Saussurea involucrata* promotes cold resilience and biomass increase in transgenic tomato plants under cold stress

**DOI:** 10.1186/s12870-021-02851-8

**Published:** 2021-02-04

**Authors:** Jianqiang Mu, Yajuan Fu, Bucang Liu, Yao Zhang, Aiying Wang, Yuxia Li, Jianbo Zhu

**Affiliations:** 1 Key Laboratory of Agricultural Biotechnology, College of Life Science, Shihezi University, Shihezi, Xinjiang, 832003 China; 2 Department of Biology, Southern University of Science and Technology, Shenzhen, 518055 Guangdong China

**Keywords:** *SiFBA5*, Calvin cycle, Photosynthesis, Cold stress, Chlorophyll

## Abstract

**Background:**

*Saussurea involucrata* survives in extreme arctic conditions and is very cold-resistant. This species grows in rocky, mountainous areas with elevations of 2400–4100 m, which are snow-covered year-round and are subject to freezing temperatures. *S. involucrata*’s ability to survive in an extreme low-temperature environment suggests that it has particularly high photosynthetic efficiency, providing a magnificent model, and rich gene pool, for the analysis of plant cold stress response. Fructose-1, 6-bisphosphate aldolase (FBA) is a key enzyme in the photosynthesis process and also mediates the conversion of fructose 1, 6-bisphosphate (FBP) into dihydroxyacetone phosphate (DHAP) and glycerol triphosphate (GAP) during glycolysis and gluconeogenesis. The molecular mechanisms underlying *S. involucrata*’s cold tolerance are still unclear; therefore, our work aims to investigate the role of FBA in plant cold-stress response.

**Results:**

In this study, we identified a cold-responsive gene, *SiFBA5*, based on a preliminary low-temperature, genome-wide transcriptional profiling of *S. involucrata*. Expression analysis indicated that cold temperatures rapidly induced transcriptional expression of *SiFBA5*, suggesting that *SiFBA5* participates in the initial stress response. Subcellular localization analysis revealed that *SiFBA5* is localized to the chloroplast. Transgenic tomato plants that overexpressed *SiFBA5* were generated using a *CaMV* 35S promoter. Phenotypic observation suggested that the transgenic plants displayed increased cold tolerance and photosynthetic efficiency in comparison with wild-type plants.

**Conclusion:**

Cold stress has a detrimental impact on crop yield. Our results demonstrated that *SiFBA5* positively regulates plant response to cold stress, which is of great significance for increasing crop yield under cold stress conditions.

**Supplementary Information:**

The online version contains supplementary material available at 10.1186/s12870-021-02851-8.

## Background

The Calvin cycle is a critical part of photosynthetic carbon fixation, providing essential compounds for plant survival [1, 2]. This process is associated with a variety of catalytic reactions and divided into three distinct phases: carbon fixation, reduction, and regeneration of the starting molecule [3]. It has been reported that plants grown in moderate light and temperature conditions can withstand more than a 50% reduction in ribulose-1, 5-biphosphate carboxylase-oxygenase (Rubisco) activity with little effect on photosynthesis. In contrast, under high light conditions, decreased Rubisco activity was related to the reduction of photosynthesis [4]. Rubisco is not always the most active enzyme during the Calvin cycle, other enzymes probably exert greater control over carbon flow during photosynthesis, specifically, sedoheptulose-1, 7-bisphosphatase (SBPase), transketolase (TK), and fructose-1,6-bisphosphate aldolase (FBA). Therefore, these enzymes are candidates for study in efforts to increase photosynthetic carbon fixation [3, 5].

FBA is a key metabolic enzyme that mediates the reversible conversion of fructose-1,6-bisphosphate (FBP) into dihydroxyacetone phosphate (DHAP) and glyceraldehyde-3-phosphate (GAP) during glycolysis and gluconeogenesis [6, 7]. In the Calvin cycle, FBA can also catalyze the condensation of sedoheptulose-1,7-bisphosphate from erythrose-4-phosphate and GAP [8, 9]. Antisense inhibition and photosynthesis modeling studies have shown that FBA may play a major role in controlling carbon metabolism rates and ribulose-1,5-biphosphate (RuBP) regeneration in the Calvin cycle [10–12]. Experiments in tobacco (*Nicotiana tabacum*) indicated that overexpression of *FBA* can promote RuBP regeneration while slightly increasing growth and biomass productivity [5]. Additionally, a study on tomato (*Solanum lycopersicum*) showed that *SlFBA7* overexpression increased the activity of several major enzymes in the Calvin cycle, as well as the net photosynthetic rate (Pn), seed size, and stem diameter [13].

FBA is also involved in plant cold-stress response. For instance, cold stress upregulates *FBA* family member *ClAldC* expression in *Codonopsis lanceolata* [14]. Interestingly, the *FBA* family responds differently to cold treatment in *Arabidopsis* and rice (*Oryza sativa*) [13, 15]. Despite extensive research on several model plants and commercial crops, few studies have explored the role of *FBA* in more extreme habitats.

*S. involucrata* is a perennial alpine plant resistant to extreme cold stress [16]. Despite having a short life cycle, *S. involucrata* can accumulate substantial biomass and has evolved numerous genes specifically associated with cold tolerance [17]. Recently, transcriptome sequencing and extensive bioinformatics analysis were completed for *S. involucrata*, providing us with an opportunity to determine the specific *FBA* gene associated with *S. involucrata* cold stress tolerance [18]. In the present study, we used Agrobacterium-mediated transformation to construct transgenic lines of tomato plants to study the putative function of *SiFBA5* genes [19, 20].

## Results

### Isolation of *siFBA5* from *S. involucrata*

**Fig. 1:**
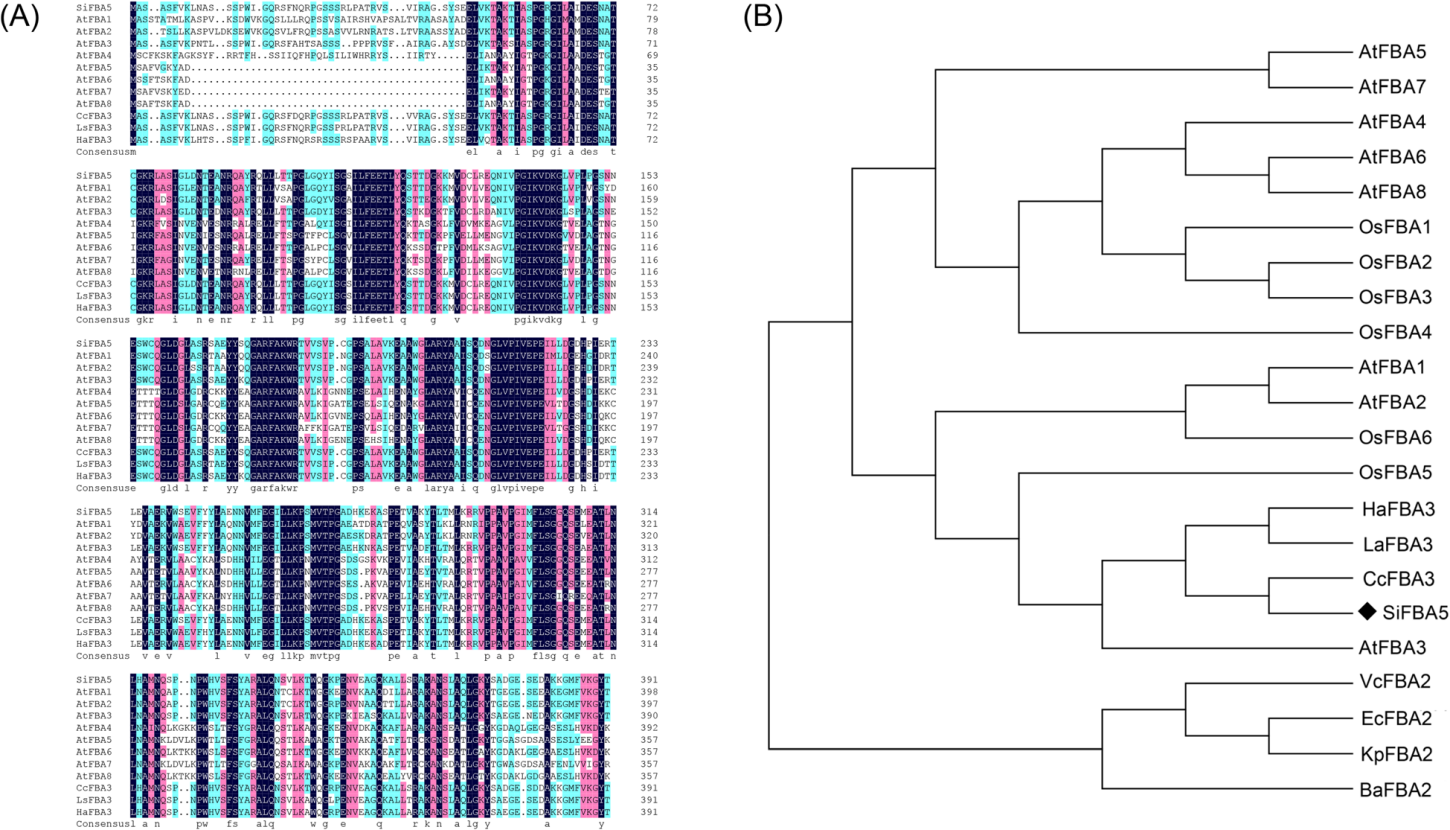
Sequence alignment and phylogeny of *SiFBA5* homologs. **a** Amino acid alignment of SiFBA5 homologs. **b** Phylogenetic assay of FBA homologous proteins across species. The following species were included: *Arabidopsis thaliana*, *Oryza sativa*, *Cynara cardunculus*, *Lactuca sativa*, *Helianthus annuus*, *Escherichia coli*, *Klebsiella pneumonia*, *Brucella abortus*, and *Vibrio celticus*

*SiFBA5* cDNA was identified from transcriptome sequencing of *S. involucrata*. Full-length cDNA was obtained using gene-specific primers [17]. Sequencing analysis revealed that the open reading frame (ORF) of *SiFBA5* cDNA (1179 bp) encodes a 392-amino-acid protein, with a molecular mass of 42.57 kDa and isoelectric point (pI) of 8.84 (Figure S1-S2). Multiple sequence alignment revealed that *SiFBA5* shared 75.2%, 74.9%, and 70.2% similarity to Compositae homologs *CcFBA3*, *LsFBA3*, and *HaFBA3*, respectively; thus, the gene was highly conserved (Fig. 1a). The gene also exhibited the canonical lysine residue of Class I aldolases involved in aldol condensation. Phylogenetic analysis indicated that *SiFBA5* is closely related to the homolog *AtFBA3*, a chloroplastic FBA (Fig. 1b). This outcome suggests that *SiFBA5* localizes to the chloroplast.

### Expression patterns of *SiFBA5*

**Fig. 2:**
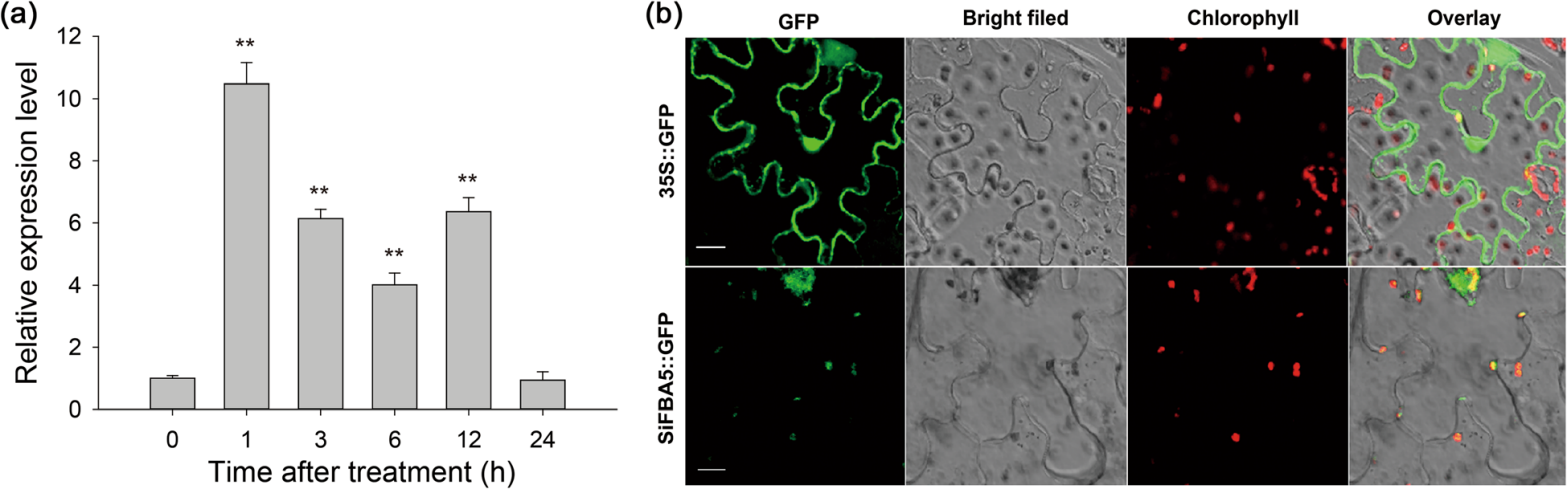
Expression patterns of SiFBA5 under cold stress and its subcellular localization. **a** qRT-PCR analysis of *SiFBA5* in *S. involucrata*. Means ± SD was obtained from three biological replicates. *, *P* < 0.05; **, *P* < 0.01. **b** Subcellular localization of *SiFBA5* in tobacco epidermal cells. *SiFBA5-GFP* and empty control vector (*35S::GFP*) were transiently expressed in tobacco epidermal cells. Fluorescent images were taken by confocal microscopy after *Agrobacterium*-mediated infiltration for 48 h. Upper row, the control panel of *35S::GFP*, lower row, *35S::SiFBA5-GFP*. From left to right: channels of GFP fluorescence, bright field, chloroplast autofluorescence and an overlay of all three. Scale bar = 20 μm. One-way ANOVA was used to compare the statistical difference between measurements (*P* < 0.05)

To investigate how *SiFBA5* responds to cold stress, *S. involucrata* rosettes at the five-leaf stage were exposed to 4 °C for 1, 3, 6, 12, and 24 h. After 1 h, the relative expression of *SiFBA5* increased 10-fold, before gradually declining (Fig. 2a). This rapid and strong induction of expression suggested that *SiFBA5* participates in the initial response to cold stress.

### Subcellular localization of *SiFBA5*

WoLF PSORT (https://wolfpsort.hgc.jp/), YLoc (www.multiloc.org/YLoc), and MultiLoc2 (http://www-bs.informatik.uni-tuebingen.de/Services/MultiLoc2) predicted that *SiFBA5* subcellular localization was in the chloroplast. To test the robustness of this prediction, we constructed a SiFBA5-GFP fusion protein and used confocal microscopy to assay its presence in *Nicotiana benthamiana* leaf cells. We confirmed that SiFBA5-GFP was localized to the chloroplast (Fig. 2b). Therefore, *SiFBA5* likely participates in the Calvin cycle and may be related to downstream gene regulation during photosynthetic carbon flux.

### Cold stress responses of transgenic *SiFBA5* overexpression lines

**Fig. 3:**
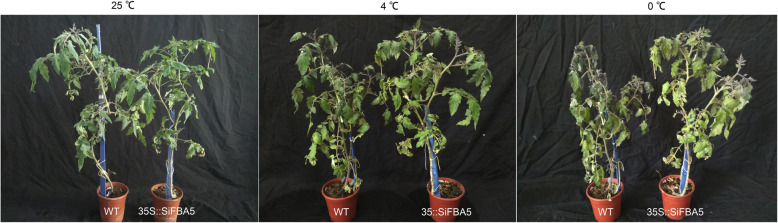
Stress responses of *SiFBA5* transgenic plants under cold. Nine-week-old WT and *SiFBA5* transgenic plants were subjected to either normal temperatures (25 °C) or cold stress conditions (4 °C and 0 °C) for 24 h before taking pictures

To reveal the functional roles of *SiFBA5*, we constructed tomato overexpression lines under the control of the 35S promoter. Analysis with PCR verified transgene integration. Under normal temperature conditions (25 °C), growth did not differ between overexpression lines and wild type, suggesting that excess *SiFBA5* does not cause an aberrant phenotype (Fig. 3). However, during cold tolerance assays (4 °C), wild-type plants exhibited thinning and drooping leaves, along with bent side-branches, whereas overexpressing *SiFBA5* plants did not. Furthermore, at 0 °C, wild-type leaves curled and deepened in color, while transgenic leaves were no longer viable (Fig. 3).

### Physiological changes in transgenic plants under cold stress

**Fig. 4:**
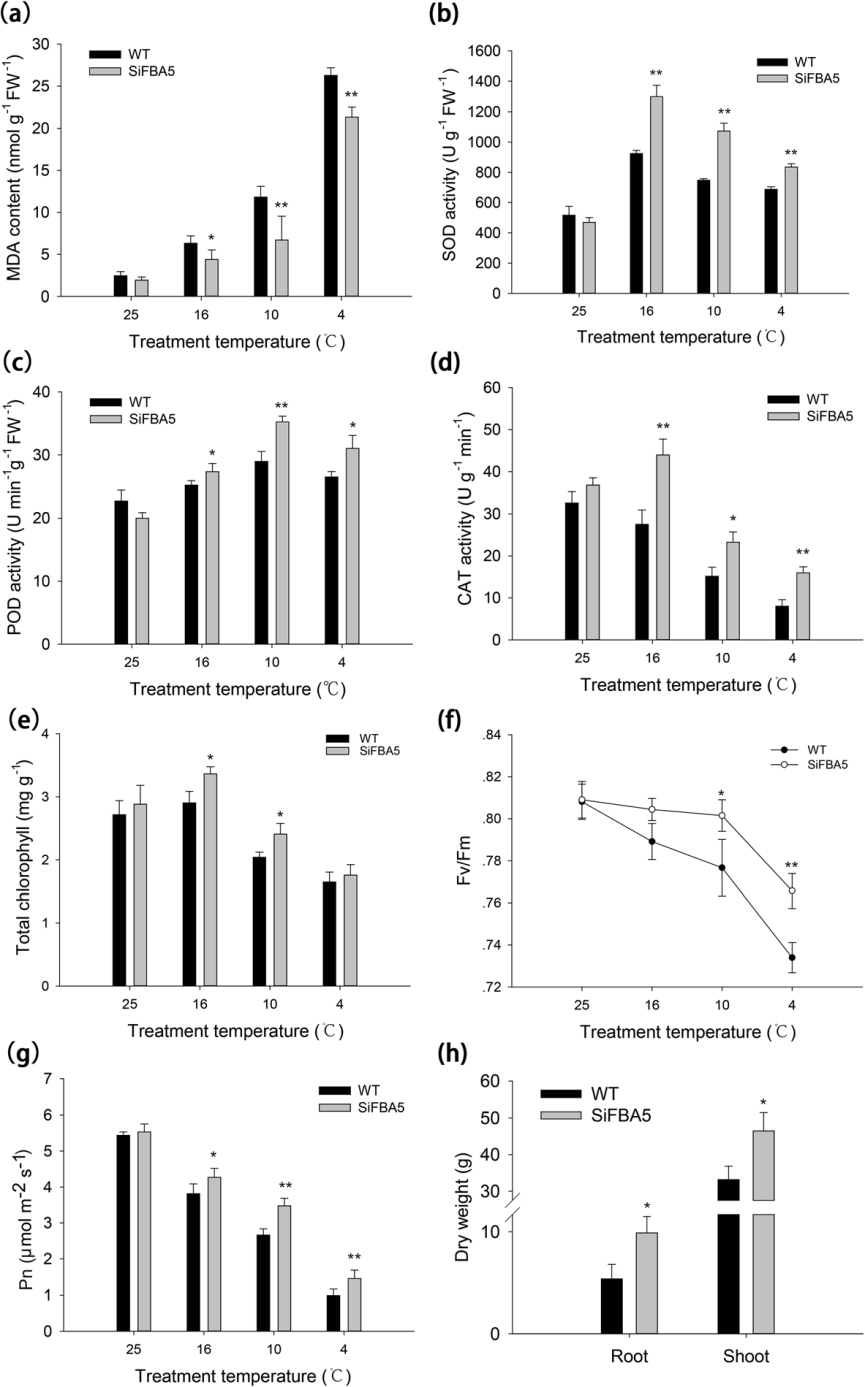
Determinations of various physiological parameters and biomass production in WT and transgenic tomato plants. **a**-**e** Physiological analysis of WT and *SiFBA5* transgenic plants under normal temperature (25 °C) and cold stress (16, 10, and 4 °C), **a** malondialdehyde (MDA), **b** superoxide dismutase (SOD), **c** peroxidase (POD), **d** catalase (CAT), and **e** total chlorophyll content. **f** Maximal efficiency of PSII photochemistry (Fv/Fm), **g** net photosynthetic rate (Pn), **h** biomass. Mean ± SD was obtained in one experiment with three biological replicates. Asterisks indicate significant differences between WT and transgenic plants (*P < 0.05; **P < 0.01). One-way ANOVA was used to compare the statistical difference between measurements (*P* < 0.05)

Cold stress causes the overproduction of reactive oxygen species (ROS) and oxidative damage in plant cells. Under cold stress conditions (4 °C), the overexpression lines exhibited significantly less malondialdehyde (MDA) accumulation than wild type (*P* < 0.05) (Fig. 4a). Since MDA is the product of ROS-induced lipid peroxidation, this outcome indicated that the plants were subject to less oxidative stress when *SiFBA5* was overexpressed. We then assayed the activities of three major antioxidant enzymes (superoxide dismutase, SOD; catalase, CAT; and peroxidase, POD) to investigate the cause of this change. There was no significant difference in SOD activity between transgenic and wild-type plants under normal conditions, but under cold stress, SOD activity was significantly enhanced (*P* < 0.05) (Fig. 4b). Additionally, we found that POD activity increased significantly in the transgenic lines compared with the wild type under cold stress (*P* < 0.05) (Fig. 4c). As temperatures decreased, the corresponding decrease in CAT activity was more pronounced than the decreases in SOD and POD activity. Transgenic and wild type plants again differed significantly, with less CAT activity in the former than in the latter (*P* < 0.05) (Fig. 4d). Our results suggest that *SiFBA5* overexpression inhibits ROS damage under cold stress by both decreasing MDA accumulation and enhancing antioxidant enzyme activity.

### Effects of *SiFBA5* on photosynthetic and chlorophyll fluorescence factors

We determined changes in net photosynthetic rate (Pn) to delve deeper into how cold stress alters photosynthetic efficiency. Our findings showed that transgenic plants and wild-type plants did not differ significantly in Pn under normal conditions. However, at 10 °C and 4 °C, overexpression lines displayed significantly higher Pn than wild type (*P* < 0.01) (Fig. 4g). In line with this observation of elevated photosynthesis under *SiFBA5* overexpression, shoot and root dry biomass were respectively 84.34 and 40.14% greater in transgenic plants than in wild type (*P* < 0.01) (Fig. 4h). We also observed significantly higher total chlorophyll content in overexpression lines than in wild type at 16 °C and 10 °C (*P* < 0.05) (Fig. 4e). Finally, we measured the maximum efficiency of PSII photochemistry (Fv/Fm), which reflects PSII photoinhibition. In the *SiFBA5* overexpression lines, Fv/Fm was greater than in wild-type plants (*P* < 0.05) (Fig. 4f), indicating that cold stress did not damage the PSII complex in transgenic plants.

### *SiFBA5* overexpression effects on other Calvin-cycle enzymes

**Fig. 5:**
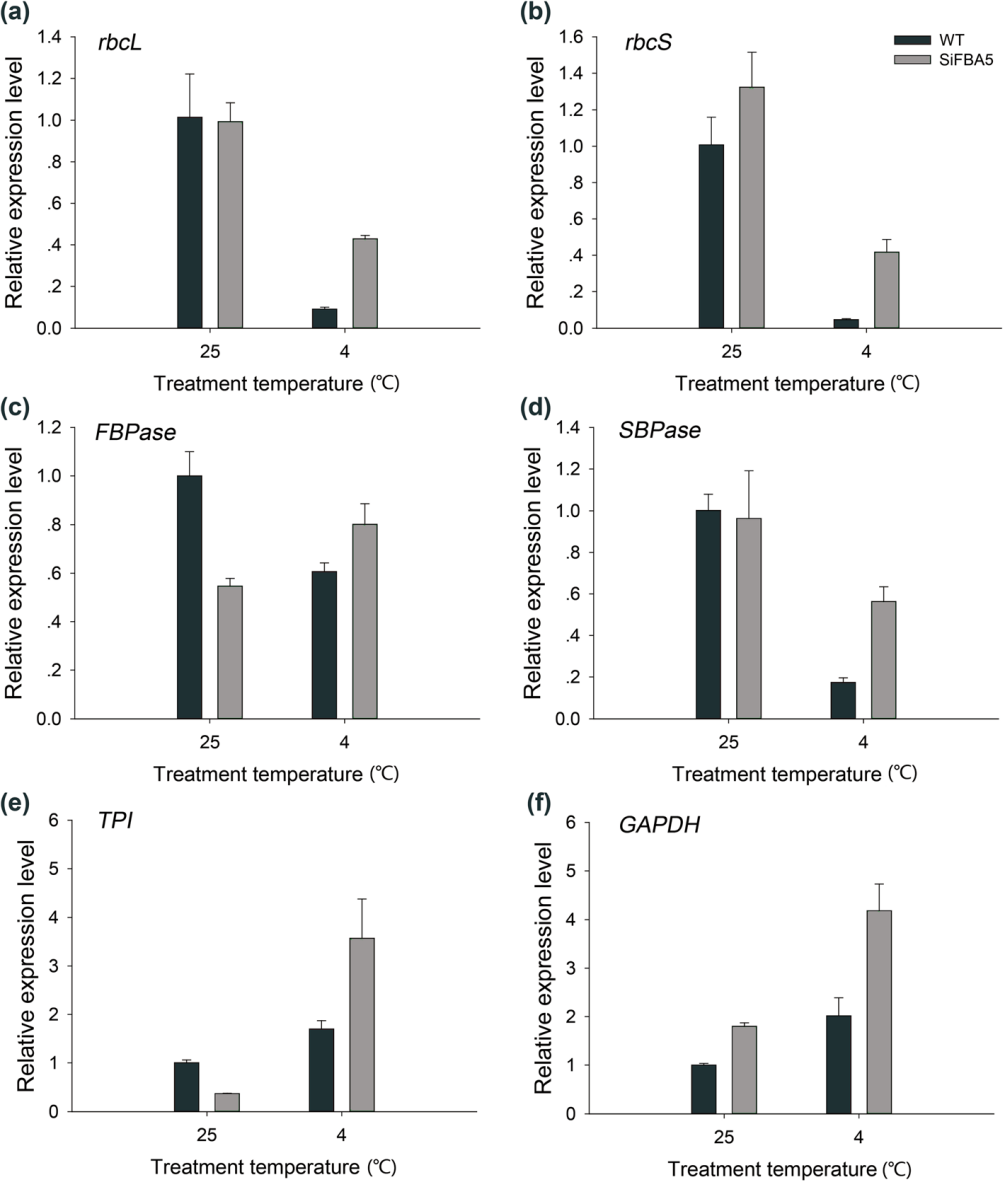
Transcriptional expression analyses of primary Calvin-cycle enzyme encoding genes in WT and transgenic tomato plants. **a** *rbcL* (Gene ID:101265242), **b** *rbcS* (Gene ID:543973), **c** *FBPase* (Gene ID:101264273), **d** *SBPase* (Gene ID:100316873), **e** *TPI* (Gene ID:778306), and **f** *GAPDH* (Gene ID:100736499). Values are mean ± SD (*n* = 3). One-way ANOVA was used to compare the statistical difference between measurements (*P* < 0.05)

We analyzed the expression of several enzymes involved in photosynthesis regulation: *Rubisco, SBPase*, *fructose-1,6-bisphosphatase* (*FBPase*), *glyceraldehyde-3-phosphate dehydrogenase* (*GAPDH*), and *triose-3-phosphate isomerase* (*TPI*). Transgenic lines showed significantly higher expression of both *rbcL* and *rbcS* (Rubisco large and small subunits) than wild type at 4 °C (*P* < 0.05) (Fig. 5a, b). Additionally, *SBPase*, *FBPase*, *GAPDH*, and *TPI* expression was significantly higher in transgenic lines than in wild type (*P* < 0.05) (Fig. 5c-f).

## Discussion

Plants are sessile and cannot avoid damage in their environment by moving, so they have evolved a series of genes to respond to stress. A previous study found that the use of Agrobacterium-mediated transformation of *Persian walnut (Juglans regia)* somatic embryos can enhance the resistance response of the plants and contribute to better appearance of the kernels for consumers [21–23]. Freezing injury is a major cause of crop loss and a limiting factor. There are some studies regarding cold hardiness mechanisms and methods of cold adaptation in walnut [24–26].

Accordingly, this study is designed to exploit the *FBA* gene from *S. involucrata* and transfer it into tomato plants. Fructose-1,6-bisphosphate aldolase is critical to carbon metabolism in plants, playing a role in processes such as glycolysis, gluconeogenesis, pentose phosphate pathway, and the Calvin cycle [7]. Studies in multiple plant species have amply demonstrated the critical role of *FBA* in regulating plant stress-tolerance, growth, and development [5, 14, 27, 28]. To the best of our knowledge, the present study is one of the first studies to examine *FBA* function in a plant species endemic to an extreme habitat, specifically *S. involucrata*, which can survive even at temperatures below − 21 °C [29]. Transcriptome analysis of *S. involucrata* identified 15 *FBA* gene transcripts, three of which were upregulated in response to cold stress (data not shown). We selected *SiFBA5* here for further analysis. A bioinformatics analysis revealed that *SiFBA5* proteins are highly similar to FBA homologs in other species. Consistent with the low-temperature transcriptome profile, cold stress significantly increased *SiFBA5* transcript abundance. Phylogenetic analysis of *SiFBA5* revealed highly homologous relationships with chloroplast FBAs in plants such as *Arabidopsis*, rice, tobacco, and tomato [5, 13, 15]. In higher plants, FBAs have two isozymes: cytosolic and chloroplast [15], the latter is involved in the Calvin cycle. In potatoes, suppression of chloroplast FBA inhibits photosynthesis [30]. Additionally, the SiFBA5-GFP fusion protein was localized to the chloroplast (Fig. 2b). Our *SiFBA5*-overexpression transgenic lines revealed improved photosynthetic efficiency and cold tolerance compared with wild-type plants under low-temperature exposure.

We also successfully identified some of the physiological processes involved in the cold stress response, such as increased MDA production. Therefore, we tested the MDA content in wild type and *SiFBA5*-overexpression transgenic lines under cold stress, which revealed a decrease in the MDA content of *SiFBA5*-overexpression transgenic lines relative to wild type (*P* < 0.05). These results indicated that *SiFBA5*-overexpressing plants had lower oxidative stress (Fig. 4a) [31]. Furthermore, under cold stress, transgenic plants had significantly higher SOD, POD, and CAT activity than in the wild type. These three enzymes are part of the antioxidant defense system that evolved in plants to counteract ROS-induced oxidative damage (e.g., lipid peroxidation) [32]. Given that abiotic stress increases the activity of these enzymes; we speculated that overexpression of *SiFBA5* can eliminate excess ROS from cells, offering protection against oxidative damage.

Low temperatures decrease plant capacity to use light energy and increase photoinhibition in cold-sensitive plants [33]. Some data suggest that photosynthesis is inhibited through reduced aldolase expression [10]. Another study found that *AtFBA* overexpression specifically promoted RuBP regeneration, CO_2_ fixation efficiency, and photosynthetic rate in transgenic tobacco [5]. Our results demonstrated that *SiFBA5* overexpression lines under cold stress constitutively elevated *FBA* expression in chloroplasts and promoted Rubisco expression, which enhanced RuBP regeneration, leading to accelerated RuBP metabolism. These changes then promote the entire photosynthetic pathway. Variation in the individual FBA of transgenic plants showed that the regenerative capacity of the C3 cycle places limitations on photosynthetic rate [3].

Cold stress decreases Fv/Fm, making the latter an important variable in screening for cold tolerance [34]. Our study confirmed that cold stress reduces Fv/Fm, resulting in plants that cannot efficiently use excitation energy and thus exhibit reduced PSII photochemical efficiency. However, transgenic plants had higher Fv/Fm than wild type under cold stress. The ability to sustain proportionately higher Fv/Fm could enhance photosynthesis after a cold-stress-induced slowdown. We also found that overexpression of chloroplast *FBA* slightly increased Pn under cold stress, but not under normal temperatures. Thus, prolonged exposure to cold stress appeared to reduce *SiFBA5* transcription, but the overexpression of *SiFBA5* partially compensated for FBA loss, which is in line with data from potatoes [30] and tomatoes [35].

## Conclusion

We successfully cloned *SiFBA5* from *S. involucrata* and identified the gene product as an aldolase. Overexpressing *SiFBA5* in tomato alleviated cold-stress-induced cellular damage, improved cold tolerance, and improved photosynthetic capacity under cold stress conditions. Our data provide a theoretical foundation and experimental basis for using *SiFBA5* in the development of *S. involucrata* germplasm resources. Moreover, the gene is a strong candidate for genetic engineering to increase crop production and stress resistance in cold climates.

## Methods

### Plant materials and treatments

*S. involucrate* seeds were a gift from Zhaosu County Forestry Bureau, Yili Prefecture, Xinjiang. Seedlings of *S. involucrata* were cultured using plant tissue culture technology and placed in a glasshouse at 19 °C with a 16 h light/8 h dark photoperiod.

*Nicotiana benthamiana* seeds were grown in pots with 3:1 peat moss and vermiculite soil (v/v) under a photoperiod of 12 h light at 26 °C/12 h dark at 22 °C, with ~ 50% relative humidity.

Tomato (Yaxin 87–5) seeds were surface-sterilized in 75% ethanol for 1 min, 2% v/v sodium hypochlorite for 15 min, and rinsed six times with sterile distilled water. Seeds were sown on 1/2 Murashige and Skoog (MS) medium. For a week, seeds were incubated at 25 °C under a 16 h light/8 h dark cycle with fluorescent lights. Cotyledon explants of 7-d-old seedlings were used for *Agrobacterium* transformation. For cold treatment, rosettes at the five-leaf stage were exposed to 4 °C for 1, 3, 6, 12, and 24 h, respectively. All tissue samples were immediately frozen in liquid nitrogen and stored at − 80 °C.

### RNA isolation and qRT-PCR

Total RNA was isolated with TRIzol from *S. involucrat*e and tomato, following manufacturer protocol. PrimeScriptTM RT reagent Kit (TaKaRa, China) was used for first-strand cDNA synthesis. Quantitative real-time PCR (qRT-PCR) was performed in the Light Cycler 480 system (Roche Diagnostics, Germany) using LightCycler® 480 SYBR Green I master mix (Roche Diagnostics). The thermocycling protocol was 40 cycles of 95 °C for 10 s, 60 °C for 20 s, and 72 °C for 30 s. Relative expression was calculated with the 2^-ΔΔCt^ method. Each expression profile was independently verified in triplicate. qRT-PCR primers are shown in Supplementary Table S1. We used *S. involucrat* cDNA to amplify the *FBA* gene and cDNA from tomato to detect the expression of related genes. The experiment is represented as means ± SE of three replications.

### Isolation of *SiFBA5* gene from *S. involucrata*

The *SiFBA5* gene sequence was obtained from *Saussurea*KBase (http://www.shengtingbiology.com/SaussureaKBase/index.jsp.). Putative full-length SiFBA5 cDNA and genomic DNA were amplified using gene-specific primers SiFBA5-F and -R (Supplementary Table S1). *SiFBA5* cDNA was ligated into the pMD®19-T simple vector (TaKaRa, China) and *E. coli* DH5α competent cells (TransGen, China); successful cloning was verified via Sanger sequencing (BGI, Beijing, China). All subsequent constructs were made using this clone as a template. *SiFBA5* gene homologs were identified using BlastP in NCBI (https://blast.ncbi.nlm.nih.gov/Blast.cgi). Multiple sequence alignments were performed in ClustalX2 with default parameters and DNAMAN. Alignments were then adjusted for constructing the phylogenetic tree in MEGA 5.0.

### DNA constructs

The coding region of *SiFBA5* was amplified using primers G-SiFBA5-F and -R, with appropriate restriction sequences (Table S1). PCR products were digested with *BamH* I and *Sal* I, then ligated with the multiple cloning site (MCS) of *pCAMBIA*2300 binary vector, containing *GFP* as the reporter under the control of *cauliflower mosaic virus* 35S (*CaMV* 35S) promoter [36]. The construct for *SiFBA5* subcellular location was generated through the in-frame fusion of complete *SiFBA5* CDS (stop codon TAG deleted) with *GFP*. To generate the overexpression construct, *SiFBA5* ORF was cloned into the *pCAMBIA*2300 vector under the control of the *CaMV* 35S promoter. The insert was released from pMD19-T-*SiFBA5* through *Sma* I and *Sal* I digestion, and then ligated into *pCAMBIA*2300 MCS. Constructs were introduced into *Agrobacterium* strain *GV3101* through electroporation using the GENE PULSER II system (Bio-Rad, Hercules, CA, USA).

### Plant transformation

The *35S::SiFBA5* vectors were introduced into a tomato for overexpression lines, respectively. Select sterile, robust tomato seedlings with fully expanded cotyledons, cut off the two ends of the cotyledons, and then divide them into two from the middle of the leaves. Use the leaf discs as genetically transformed explants, and spread them on MS + 2 mg/L 6-BA+ 0.15 mg/L IAA medium with the backside down, dark culture for 2 days. Transformation into the tomato, with cotyledons as explants, followed published protocols [37]. Callus induction and shoot regeneration were induced. Next, inoculated explants were screened on MS medium supplemented with different concentrations of plant growth regulators and antibiotics. Shoots were regenerated on a selective medium containing kanamycin (50 mg L^− 1^). Rooted kanamycin-resistant primary transformants (5–10 cm high) were transferred to potting soil; S*iFBA5* presence was confirmed via PCR. The *35S::SiFBA5-GFP* vectors were introduced into tobacco for subcellular location experiments, the third to fifth youngest leaves of the three- to four-week-old *Nicotiana benthamiana* (approximately 8–10 leaf stage) was injected *with Agrobacterium*-expressing GFP-fusion protein using a 1 mL syringe. Following 2–3 d incubation in growth chambers, a laser scanning confocal microscope (Zeiss, Germany) was used to image GFP fluorescence in tobacco leaves.

### Stress treatment

Stress treatments were initiated after plants were grown for 9 weeks in the soil at 25 °C under a 16 h/8 h (light/dark) photoperiod. To investigate the role of *SiFBA5* in cold tolerance, wild-type and transgenic tomato plants were incubated (Percival Scientific, USA) for a week under a 12/12 h light (25 °C)/dark (22 °C) cycle with moderate light intensity and 50–60% relative humidity. Then, wild-type and transgenic tomato plants were subjected to cold stress at 16, 10, and 4 °C, each for 3 d. Subsequently, plants were placed overnight in a chamber set to 0 °C.

### Physiological measurements

Physiological traits were measured at different stages after cold stress treatments. Analysis of stress-induced oxidative damage used an MDA assay as previously described [38]. The nitro blue tetrazolium (NBT) photoreduction method was used to estimate SOD activity [39]. Next, POD activity was determined through monitoring increases in absorbance at 470 nm during guaiacol oxidation [40]. Finally, CAT activity was assayed through monitoring decreases in H_2_O_2_ absorbance (240 nm) within 1 min [41]. Total chlorophyll contents were measured following a previous method with some modifications [33]. We weighed out 0.1 g of leaf tissue into pieces, total chlorophyll was extracted using ethanol: acetone: H_2_O (4.5:4.5:1) and analyzed by UV spectrophotometry.

### Gas-exchange and chlorophyll fluorescence

Net photosynthesis and stomatal conductance were detected using the portable photosynthesis system LI-6400 (LI-COR, USA). Chlorophyll fluorescence was measured with a Mini PAM chlorophyll fluorometer (Waltz, Germany) [42].

### Statistical analysis

All data are represented as means ± SE of three replications. SPSS_13.0_ software (SPSS, Chicago, USA) was used to compare MDA content, SOD activity, POD activity, CAT activity, or relative gene expression in the control and transgenic lines. One-way analysis of variance followed by Tukey’s honestly significant difference (*P* < 0.05 and *P* < 0.01) multiple comparison tests were used to determine the significant differences. Error bars in all figures represent standard deviations from the mean. SigmaPlot_12.0_ was used to generate figures.

## Supplementary Information

**Additional file 1: Figure S1.** Nucleotide and deduced amino acid sequences of SiFBA5. The numbers of Nucleotide and amino acid are shown on the left.

**Additional file 2: Figure S2.** PCR identification of transgenic tomato line. (a) PCR assay of the transgenic plants; (b) RT-PCR assay of the transgenic plants. M: DNA Marker; Number means different transgenic lines; “+” The positive plasmid; WT: wild-type tomato plant.

**Additional file 3: Table S1.** List of primers used in this study.

## Data Availability

All data generated or analyzed during this study are included in this published article and its supplementary information files. Also, original images are available from the corresponding author upon reasonable request (Jianbo Zhu, zhujianboSHZU@163.com).

## Abbreviations

qRT-PCR: Quantitative Real-Time PCR
SBPase: Sedoheptulose-1,7-Bisphosphatase
TK: Transketolase
FBA: Fructose-1,6-Bisphosphate Aldolase
FBP: Fructose-1,6-Bisphosphate
DHAP: Dihydroxyacetone Phosphate
GAP: Glyceraldehyde-3-Phosphate
RuBP: Ribulose-1,5-Biphosphate
Pn: Photosynthetic rate
MS: Murashige and Skoog
ROS: Reactive Oxygen Species
MDA: Malondialdehyde
SOD: Superoxide Dismutase
CAT: Catalase
POD: Peroxidase
MCS: Multiple Cloning Site
NBT: Nitro Blue Tetrazolium
