# Supplementary figures and images for: SiFBA5, a cold-responsive factor from Saussurea involucrata promotes cold resilience and biomass increase in transgenic tomato plants under cold stress
Source: BMC Plant Biol. 2021 Feb 4;21:75. doi: 10.1186/s12870-021-02851-8 (PMC7863501; doi:10.1186/s12870-021-02851-8)

**Figure S1**


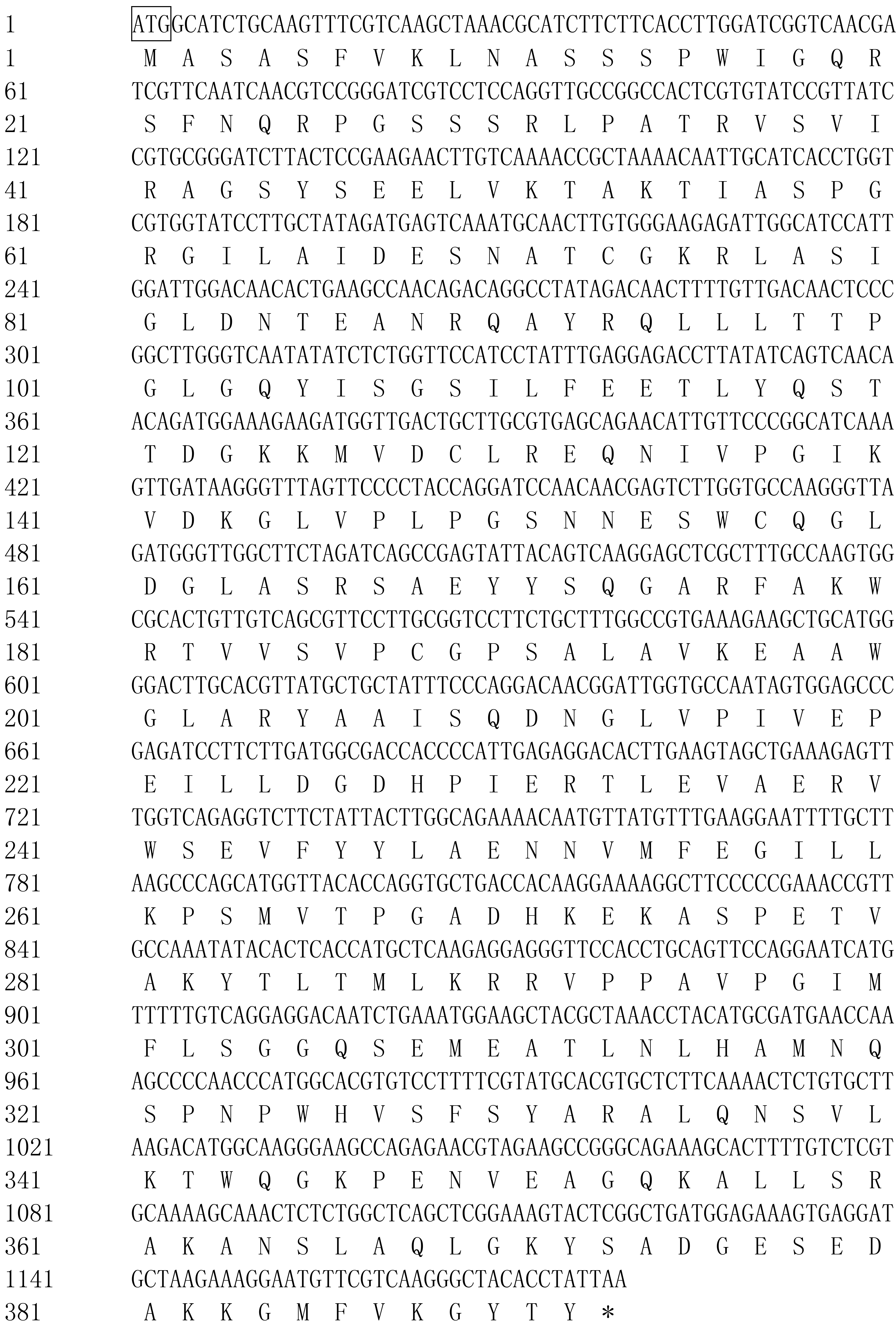

Supplement: Supplementary file 1 — Additional file 1: Figure S1. Nucleotide and deduced amino acid sequences of SiFBA5. The numbers of Nucleotide and amino acid are shown on the left. [file 12870_2021_2851_MOESM1_ESM.docx]

**Figure S2**

**
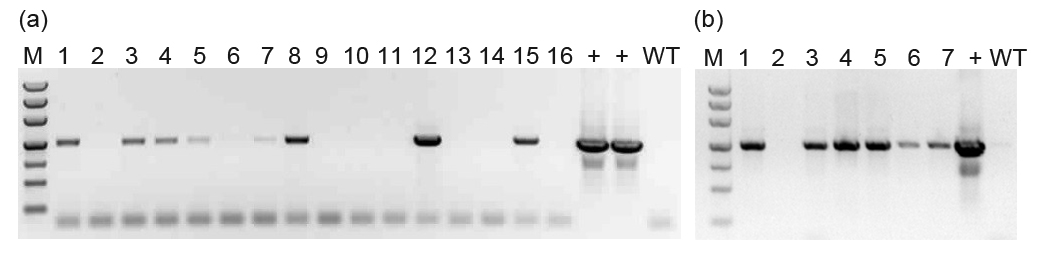
**

Supplement: Supplementary file 2 — Additional file 2: Figure S2. PCR identification of transgenic tomato line. (a) PCR assay of the transgenic plants; (b) RT-PCR assay of the transgenic plants. M: DNA Marker; Number means different transgenic lines; “+” The positive plasmid; WT: wild-type tomato plant. [file 12870_2021_2851_MOESM2_ESM.docx]
