# Supplementary material for: SiFBA5, a cold-responsive factor from Saussurea involucrata promotes cold resilience and biomass increase in transgenic tomato plants under cold stress
Source: BMC Plant Biol. 2021 Feb 4;21:75. doi: 10.1186/s12870-021-02851-8 (PMC7863501; doi:10.1186/s12870-021-02851-8)
Supplement: Supplementary file 3 — Additional file 3: Table S1. List of primers used in this study. [file 12870_2021_2851_MOESM3_ESM.docx]

Supplementary material

Table S1. List of primers used in this study.

| **Primer name** | **Primer sequence (5’–3’)** | **Purpose** |
| --- | --- | --- |
| SiFBA5(*Sma*I)_F | cccgggCTTACACACACTTCGGAAATCTT | Cloning |
| SiFBA5(*Sal*I )_R | gtcgacCTACCTTAATAGGTGTAGCCCTT | Cloning |
| SiFBA5-S(*Cla* I )_F | gaattcACACACACTTCGGAAATCTTG | Subcellular localization |
| SiFBA5-S(*EcoR*I )_R | atcgatATAGGTGTAGCCCTTGACGAAC | Subcellular localization |
| SiGAPDH_qF | TAGCAAGGATGCTCCCATGTTCGT | qRT-PCR |
| SiGAPDH_qR | AAAGGAGCAAGGCAGTTGGTTGTG | qRT-PCR |
| SiFBA5_qF | ACTCGTGTATCCGTTATCCGTG | qRT-PCR |
| SiFBA5_qR | GGCTTCAGTGTTGTCCAATCCA | qRT-PCR |
| Slactin_F | GAAATAGCATAAGATGGCAGACG | qRT-PCR |
| SlActin_R | ATACCCACCATCACACCAGTAT | qRT-PCR |
| SlrbcS1_F | AAATGGATGGGTTCCTTGCTTG | qRT-PCR |
| SlrbcS1_R | TACGCCTTCTTCGCCTCTTGA | qRT-PCR |
| SlTPI_F | TTCTGGGAGGAGACTATGGGATG | qRT-PCR |
| SlTPI_R | CCTTTGAGGTGCCTGAGCTTAAC | qRT-PCR |
| SlFBP_F | TTACATAGGTTGTCTCGTGGGTGA | qRT-PCR |
| SlFBP_R | GGTGTTCGCTGATGTATCTGCTC | qRT-PCR |
| SlSBP_F | TAAGAGCCACATTTGACAATCCTG | qRT-PCR |
| SlSBP_R | CTAACGGTGCCACTTCAAACAG | qRT-PCR |
| SlGAPDH _F | GGATGCTCCTATGTTTGTTGTTGG | qRT-PCR |
| SlGAPDH_R | ACGGTCTTCTGAGTGGCTGTGAT | qRT-PCR |
| SlrbcL_F | TGCTTCCACCTACTCCCCTTTTAC | qRT-PCR |
| SlrbcL_R | TTCTTCCTTTCAACCCCACCTGT | qRT-PCR |
| SlrbcS_F | AAATGGATGGGTTCCTTGCTTG | qRT-PCR |
| SlrbcS_R | TACGCCTTCTTCGCCTCTTGA | qRT-PCR |
